# Impact of Recreational Cannabis Legalization on Opioid Prescribing and Opioid-Related Hospital Visits in Colorado: an Observational Study

**DOI:** 10.1007/s11606-023-08195-3

**Published:** 2023-06-20

**Authors:** Christine Buttorff, George Sam Wang, Asa Wilks, Gregory Tung, Amii Kress, Dan Schwam, Rosalie Liccardo Pacula

**Affiliations:** 1 RAND Corporation, Arlington, VA USA; 2 Department of Pediatrics, Children’s Hospital Colorado, University of Colorado Anschutz Medical Campus, Aurora, CO USA; 3 RAND Corporation, Santa Monica, CA USA; 4 Department of Health Systems, Management & Policy, Colorado School of Public Health, University of Colorado Anschutz Medical Campus, Aurora, CO USA; 5 ECHO Data Analysis Center, Department of Epidemiology, Johns Hopkins Bloomberg School of Public Health, Baltimore, MD USA; 6 Sol Price School of Public Policy, Schaeffer Center for Health Policy & Economics, University of Southern California, Los Angeles, CA USA

**Keywords:** Opioid use, Cannabis, Marijuana, Claims data, PDMP, Colorado Hospital Association

## Abstract

**Background:**

Cannabis may be a substitute for opioids but previous studies have found conflicting results when using data from more recent years. Most studies have examined the relationship using state-level data, missing important sub-state variation in cannabis access.

**Objective:**

To examine cannabis legalization on opioid use at the county level, using Colorado as a case study. Colorado allowed recreational cannabis stores in January 2014. Local communities could decide whether to allow dispensaries, creating variation in the level of exposure to cannabis outlets.

**Design:**

Observational, quasi-experimental design exploiting county-level variation in allowance of recreational dispensaries.

**Subjects:**

Colorado residents

**Measures:**

We use licensing information from the Colorado Department of Revenue to measure county-level exposure to cannabis outlets. We use the state’s Prescription Drug Monitoring Program (2013–2018) to construct opioid-prescribing measures of number of 30-day fills and total morphine equivalents, both per county resident per quarter. We construct outcomes of opioid-related inpatient visits (2011–2018) and emergency department visits (2013–2018) with Colorado Hospital Association data. We use linear models in a differences-in-differences framework that accounts for the varying exposure to medical and recreational cannabis over time. There are 2048 county-quarter observations used in the analysis.

**Results:**

We find mixed evidence of cannabis exposure on opioid-related outcomes at the county level. We find increasing exposure to recreational cannabis is associated with a statistically significant decrease in number of 30-day fills (coefficient: −117.6, *p*-value<0.01) and inpatient visits (coefficient: −0.8, *p*-value: 0.03), but not total MME nor ED visits. Counties with no medical exposure prior to recreational legalization experience greater reductions in the number of 30-day fills and MME than counties with prior medical exposure (*p*=0.02 for both).

**Conclusions:**

Our mixed findings suggest that further increases in cannabis beyond medical access may not always reduce opioid prescribing or opioid-related hospital visits at a population level.

## INTRODUCTION

Cannabis legalization has become one potential policy states have implemented, in part, to help reduce the tide of harm and death caused by the evolving opioid crisis that continues to kill thousands of Americans every year.^[1–3]^ By 2022, 37 states legalized access to medical cannabis and 21 states legalized access to recreational cannabis.^[4]^

Findings on whether cannabis legalization leads to reductions in opioid consumption or resulting adverse events such as overdoses have been contradictory. Findings obtained from healthcare prescription claims data showed that increased access to cannabis is associated with lower opioid prescribing.^[5–10]^ Additional evidence suggested that the legalization of cannabis, or in some cases, allowances for dispensaries, was associated with reduced opioid-related mortality.^[11–18]^ However, Shover et al. (2019) found that by extending the years of data over which the association is analyzed, the negative association between medical-cannabis laws and opioid-related mortality not only dissipates, but reverses itself.^[19]^

Most previous studies in this area have used state-level policy changes to analyze the association between cannabis legalization and opioid use. State-level policy indicators mask the substantial variation at the local level in terms of cannabis access and availability, since most states allow local jurisdictions to opt out of retail sales.^[20,21]^ This has led some to criticize earlier findings for suffering from an aggregation bias or ecological fallacy.^[22,23]^ However, only formal randomized controlled trials can truly determine whether cannabis is a substitute for opioids in pain management.^[24]^ Very limited work at the individual level using survey or trial data from specific geographic areas showed that cannabis was not a substitute for opioid use.^[25–28]^

In the current paper, we add to the ongoing debate regarding the relationship between cannabis legalization and opioid-related harms by exploiting the geographic availability of cannabis within a single state, Colorado. Using within-state data eliminates the problem of confounding differential state policy approaches to the opioid epidemic, which are often ignored.^[17]^ While opioid mortality has risen over time, Colorado has experienced lower opioid-related mortality rates than the national average.^[29]^ Furthermore, there is often a gap in time between when a state passes a legalization law and when dispensaries actually open to the public.

Colorado is unique in that it was an early adopter of both medical (2000) and recreational cannabis (2012), where medical dispensaries could begin operating in 2010 and recreational in 2014. However, local communities allowed dispensary sales, which created a unique natural experiment in dispensary exposure. Counties that adopted medical cannabis sales prior to recreational may have more population-wide experience with cannabis use. If access to cannabis reduces opioid use and resulting sequelae, then we hypothesize that counties with no or low baseline exposure to medical cannabis should see a larger impact of exposure to recreational cannabis than counties with prior high medical exposure.

## METHODS

### Data Sources and Measures

Several data sources were used for this study that broadly cover the period January 1, 2011, through December 31, 2018. A few exceptions are noted below.

### Cannabis Dispensary Data

Information on the number of medical and recreational cannabis dispensaries per county were obtained from the Colorado Department of Revenue (DOR). The DOR data includes license number, name, and address information, making it possible to identify when the dispensary began operating for which type, since co-located medical and recreational dispensaries have different license numbers.

The state began accepting applications for medical dispensary licenses in 2010; however, the first applications were not recorded until November 2010 as it took time for Colorado to set up its system. For this reason, we limit the license data to 2011 onward, and include information only on approved dispensaries. Licensing of recreational dispensaries went into effect in January 2014, although only existing medical cannabis dispensaries were initially allowed to apply for a recreational license for the first three quarters of 2014.^[30]^ As late as 2013, nearly 50% of county jurisdictions in Colorado did not allow even medical dispensaries.

Exposure to cannabis markets is measured in three ways. First, we defined recreational exposure with an indicator for when a county had one or more new recreational stores post legalization. Second, to capture exposure to medical dispensaries, we divided counties into three groups based on their level of exposure to medical cannabis dispensaries in each quarter: no dispensary counties (50% of our sample); low (fewer than 10, 40% of our sample); and high (10 or more, 10% of our sample). For the sensitivity analysis, we stratified counties into whether they had no or any medical dispensaries at baseline. We hypothesized that the “no medical” dispensary counties are more “exposed” to the passage of legal recreational cannabis than the high baseline dispensary exposure group, and thus should see a bigger effect on opioid-related outcomes.

### Colorado Prescription Drug Monitoring Program (PDMP) Database

Legally prescribed opioids are often the gateway for many opioid addictions and still represent a key drug in physician-monitored pain management. The PDMP also captures patients with non-chronic-opioid prescriptions, who also may substitute cannabis for opioids. This database captures adjudicated pharmacy claims for all US Drug Enforcement Agency scheduled drugs, and comes from the Colorado Department of Regulatory Agencies. In 2012, the PDMP switched data vendors and the levels of reported prescriptions changed markedly. Thus, we limited the use of the PDMP data for the period 2013–2018. Because all licensed pharmacies report data directly to the PDMP on every prescription filled involving a scheduled substance, ^[31]^ the PDMP effectively represents the entire population of Colorado being prescribed an opioid; and hence, it provides us with the strongest measure of opioid prescribing patterns available. Other claims databases miss the uninsured and those paying cash. Opioids were identified using the Centers for Disease Control and Prevention’s list of outpatient opioids that are identified at the National Drug Code level.^[32]^

We construct two measures of prescribing from the PDMP data. The first is the number of 30-day fills per county resident. The second is the total morphine milligram equivalents (MME) per capita. The calculation of MME multiplies the prescription strength, the quantity dispensed, and the MME conversion factor,^[33]^ and then divides this amount by the days supplied.^[34]^ We combined overlapping and concurrent fills to assign each patient a daily MME following previous work.^[34]^ Each prescription was summed to create the total MME for the quarter and then divided by the total number of residents in the county.

### Colorado Hospital Association (CHA)

While the PDMP captures legally prescribed opioids, we know that illicit opioid markets continue to proliferate.^[3]^ For this reason, we use CHA data on inpatient and emergency department (ED) visits at general and acute care hospital facilities, allowing us to identify opioid-related visits regardless of whether the opioids were purchased legally or illegally.^[35]^ CHA’s data captures downstream effects of opioid use from all insurance types, including the uninsured.

We use inpatient discharges for the period 2011–2018, but because CHA did not begin collecting ED visits until 2013, the ED visits cover only 2013–2018. For both the inpatient and ED visits, we identify opioid-related hospital visits using the International Classification of Disease (ICD) Version 10 codes and their corresponding Version 9 codes, though we report just the Version 10 codes here for brevity. These diagnosis codes included opioid use, misuse or dependence (F11*), opioid poisonings (T400*–T404*, T406*, and T507*), and the long-term use of opioids (Z798*). We err on the side of being more comprehensive in the codes included because of the difficulty in exactly crosswalking ICD codes between Versions 9 and 10. We constructed two measures: (1) the number of opioid-related inpatient visits per capita per county, and (2) the number of opioid-related ED visits per capita per county. Both were based on the patient’s county of residence.

### Analytic Approach

Our analytic method uses recent developments in the quasi-experimental design literature for difference-in-differences designs. Recent work has shown that treatment effects can be biased when the treatment occurs at different times.^[36,37]^ In our context, counties opening recreational stores in later years may have a different treatment effect because the potency of cannabis products increased while the price decreased over time. As a result, we implement the Gardner (2021) two-stage difference-in-difference approach: (1) estimate the outcome in the never treated/pre-treated observations, controlling for time and county fixed effects; and (2) estimate the treatment variable on the difference between the treated observations and the estimated never/pre-treated trend. We use the following model:

$${y}_{it}=\beta_{0}+\beta_{1}\chi_{it}+\delta_{t}+\gamma_{i}+\varepsilon_{it}$$

Where *y*_*it*_ is the outcome in county *i* in quarter *t. X*_*it*_ is the calendar year county unemployment rate and total number of county hospital admissions, drawn from the Bureau of Labor Statistics^[38]^ and the American Health Resource File,^[39]^ respectively. We also include the categorical variable for the baseline level of medical dispensaries (none, low, high) in county *i* at quarter *t*. *δ*_*t*_ is the interaction of year * quarter fixed effects and *γ*_*i*_ are county fixed effects.

The second stage estimates the average outcome in all counties minus the estimated effect in the untreated counties to isolate the effect of initiating recreational sales in a county with the following model:

$$\left({y}_{it}-\widehat{y_{it}}\right)=\alpha_{0}+\alpha_{1}{RecExposure}_{it}$$

Where *y*_*it*_ is our opioid outcome of interest in county *i* and quarter* t*. The parameter $\alpha_{l}$ captures the impact of recreational stores opening in the county (${RecExposure}_{it}$). Standard errors are clustered at the county level. We used the *did2s* command in R to estimate the models.^[40]^ The RAND Human Subjects Protection Committee deemed this study to be exempt from human subjects protections.

## RESULTS

Over the entire period, the number of medical dispensaries per capita did not change markedly, hovering around 1 per 10,000 residents per county per quarter, or about 8 dispensaries per county per quarter (Table 1). Recreational dispensaries increased from 3.6 per county per quarter in 2014 to 8.5 in 2018. While total MME and number of 30-day fills generally decreased from 2015 onward, rates of opioid-involved inpatient and ED visits both rose through 2017.

**Table 1:** Average Values of Measures for Cannabis Exposure and Opioid Outcomes at the County-Quarter Level, by Year

		**Measures of cannabis market**				**Opioid outcomes**			
		Number of medical dispensaries	Medical dispensaries per 10k	Number of recreational dispensaries	Recreational dispensaries per 10k	ED Visits per 10k (CHA)	Inpatient visits per 10k (CHA)	Total MME per county per capita (PDMP)	Average number of 30-day fills per 10k
**Full **period** (2011–2018)**	Mean	8.0	1.0	4.1	1.2	4.9	4.1	175.2	1153.8
	SD	29.2	1.6	16.8	3.4	6.1	3.4	90.0	564.5
**2011**	Mean	7.8	1.0				2.8		
	SD	28.3	1.9				2.6		
**2012**	Mean	7.9	1.1				3.3		
	SD	28.5	1.9				2.7		
**2013**	Mean	8.1	1.1			2.6	3.3	195.9	1047.7
	SD	28.9	1.9			3.1	2.9	109.5	569.7
**2014**	Mean	7.9	1.1	3.6	0.8	3.3	3.6	186.8	1127.1
	SD	27.9	1.7	15.3	1.7	4.5	3.1	83.3	529.9
**2015**	Mean	8.2	1.0	6.0	1.9	4.7	4.2	192.7	1316.1
	SD	29.2	1.6	19.2	4.0	6.0	3.7	89.8	620.2
**2016**	Mean	8.3	0.9	7.0	2.3	6.4	5.5	186.6	1287.4
	SD	31.1	1.5	21.2	4.4	7.3	4.0	93.2	608.9
**2017**	Mean	8.1	0.9	7.8	2.4	6.7	5.3	159.0	1149.9
	SD	30.4	1.3	23.4	4.6	7.1	3.5	78.1	531.9
**2018**	Mean	7.8	0.8	8.5	2.6	5.8	4.7	130.0	994.5
	SD	29.4	1.1	24.1	5.1	6.6	3.7	60.9	439.1

**Figure. 1:**
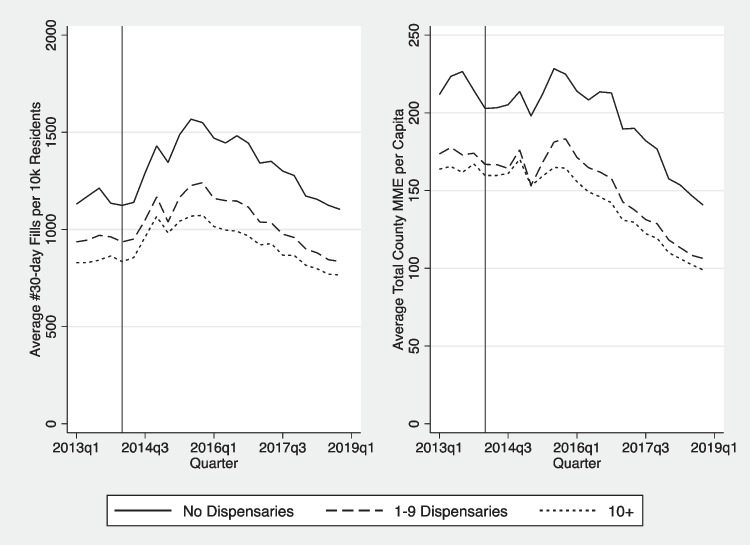
Trends in number of 30-day fills and total MME, by level of medical-cannabis dispensary exposure, 2011–2018. MME, milligram morphine equivalents. The vertical line represents the start of legal recreational sales in January 2014, when recreational dispensaries were allowed to open. Counties are categorized according to the number of medical-cannabis dispensaries in Quarter 3, 2012. The dotted bars indicate the level of opioid-related visits in the highest exposure medical-cannabis counties; the dashed lines show the outcomes in counties with 1–9 medical dispensaries; and the solid lines show trends in counties with no medical dispensaries as of November 2012, prior to legalization of recreational cannabis.

**Figure. 2:**
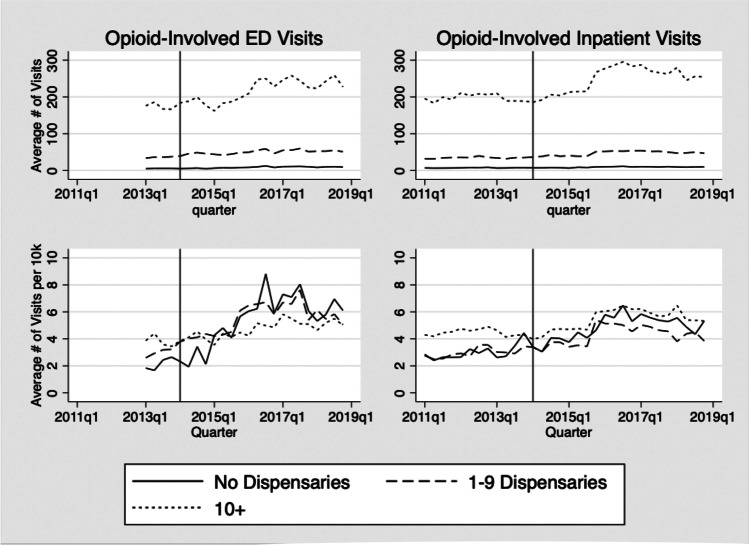
Trends in opioid-involved ED and inpatient visits, by level of medical-cannabis dispensary exposure, 2011–2018. The vertical line represents the start of legal recreational sales in January 2014, when recreational dispensaries were allowed to open. Counties are categorized according to the number of medical dispensaries in Quarter 3, 2012 (none; 1–9; and 10+). The dotted lines indicate the level of opioid-related visits in the highest exposure medical cannabis counties; the dashed lines show the outcomes in counties with 1–9 medical dispensaries; and the solid lines show trends in counties with no medical dispensaries as of November 2012, prior to legalization of recreational cannabis.

For all opioid-related outcomes, the trends do not indicate an immediate drop in opioid prescribing or opioid-related hospital visits post legalization. Figure 1 shows the downward trend in number of 30-day fills and MME after 2015 which is consistent across levels of baseline medical dispensary exposure. Counties with no prior medical exposure have higher levels of opioid use. Figure 2 shows trends in the quarterly average number of opioid-involved ED visits (left panel) and inpatient visits per 10,000 residents (right panel) for the study period. In the upper panel, areas with higher levels of medical dispensary exposures have higher absolute levels of opioid-involved visits compared to counties with relatively low or no exposure, although the trends are generally consistent. When we adjust for population within the county (lower panel), the differences in levels shrink; however, the counties with no baseline medical exposure have a generally higher level of opioid-related ED visits in later years.

Table 2 provides the primary findings of the impact of exposure to cannabis dispensaries on opioid-related outcomes. We find a significant reduction in the number of 30-day fills after legalization of recreational cannabis of −117.6 fills per county per quarter. Counties with exposure to one or more recreational cannabis dispensary had −0.8 fewer hospitalizations per 10,000 residents per quarter (*p*=0.03), than counties with no recreational cannabis. For a sense of scale, there are approximately 715,000 Denver residents, which would imply 57 fewer opioid related inpatient visits per quarter in Denver (700k/10k * 0.8 = 57). We found no statistically significant direct effect of recreational dispensaries on either opioid-involved ED visits or total MME prescribed per resident per county per quarter.

**Table 2:** Regression Results of Cannabis Dispensary Exposure on Opioid-Related Outcomes

Dependent variable	*n*	Estimate	Standard error	*p*-value
Number of 30-day fills per 10k residents	1536	−117.6	29.4	<0.01
Total opioid MME per capita	1536	−10.7	6.7	0.11
ED opioid per 10k residents	1536	−1.3	0.9	0.14
Inpatient opioid per 10k residents	2048	−0.8	0.4	0.03

Unit of analysis is the county-quarter. *MME*, morphine milligram equivalent. All models include county fixed effects and a time-varying measure on the number of medical dispensaries per county per quarter

^***^Significance at the 0.01% level; **significance at the 1% level; *significance at the 5% level; ^+^significance at 10% level. All models estimated using the did2s package in R with standard errors clustered at the county

In the sensitivity analysis, we stratified counties by whether they had previous exposure to medical cannabis, under the hypothesis that counties with no experience with medical should experience greater reductions in opioid use following recreational cannabis legalization, than counties with previous medical dispensary exposure (Table 3). We found that the number of 30-day fills dropped −130.3 compared with −120.9 in counties with prior exposure, and the MME drops −33.8 compared with −7.5 in counties with prior medical exposure (both significant at the 5% level).

**Table 3:** Sensitivity Analysis—County Stratification by Prior Medical Dispensary Exposure

**Dependent variable**	**Estimate**	**SE**	***p*-value**
**No medical prior to legalization + never treated**			
**Opioid 30-day fills per 10k residents**	−130.3	54.3	0.02
**Total opioid MME per capita**	−33.8	13.9	0.02
**ED opioid per 10k residents**	0.1	2.1	0.98
**Inpatient opioid per 10k residents**	−0.5	0.8	0.52
**Medical prior to legalization + never treated**			
**Opioid 30-day fills per 10k residents**	−120.9	31.4	<0.01
**Total opioid MME per capita**	−7.5	6.6	0.25
**ED opioid per 10k residents**	−2.0	0.9	0.02
**Inpatient opioid per 10k residents**	−0.8	0.4	0.06

Unit of analysis is the county-quarter. *MME*, morphine milligram equivalent. Counties are stratified by whether they had a medical dispensary in 2012, prior to legalization of recreational cannabis. The “never-treated” are counties that never allow medical nor recreational dispensary sales and are used as the comparison group for both models. All models include county fixed effects. ***Significance at the 0.01% level; **significance at the 1% level; *significance at the 5% level; ^+^significance at 10% level. All models estimated using the did2s package in R with standard errors clustered at the county level

## DISCUSSION

With Colorado’s rapid growth in recreational dispensaries, we find a statistically significant drop in prescribing of −117.6 fills per county per quarter, while the total MME per resident remained unchanged. We found a −0.8 per 10,000 residents decrease in opioid-related inpatient visits, but no statistically significant impact on ED visits.

The PDMP data capture legally prescribed opioids. The MME may not have changed if patients who required access to cannabis for medicinal purposes had sufficient access under the previous medical cannabis policy, even in counties where medical dispensaries were not available. The reduction in fills may be among patients only occasionally filling opioid prescriptions whereas overall MME may stay more constant if the higher MMEs are coming from chronic pain patients.

The direction of effects we find for ED and inpatient visits is consistent with the hypothesis that cannabis may substitute for opioids (though ED visits are not statistically significant), but contrasts with two recent studies using sub-state data to assess the relationship between cannabis availability and opioid-related hospitalizations. Freisthler et al. (2020) use sub-state data in California and found dispensaries were associated with increased opioid-related hospitalizations.^[41]^ Liang and Shi (2019) find that recreational dispensaries are positively associated with opioid use disorder diagnoses coded during an inpatient stay, but no association was found for medical dispensaries.^[42]^ Due to the shift from using ICD9 to 10 during the study periods, we cannot rule out there is some measurement error in the outcomes between studies due to the exact codes used.

While the main models control for medical exposure, we also conducted a sensitivity analysis stratifying on whether a county had prior medical exposure to assess the differential impact of recreational cannabis in counties with no prior medical exposure. We find that counties with no prior medical exposure had greater decreases in both 30-day fills and MME than counties with prior exposure to medical dispensaries. The changes in ED or inpatient visits for opioid-related outcomes were generally not significant, though there was a statistically significant decline in ED visits for the counties with prior medical-dispensary exposure. The finding for the opioid prescribing outcomes is consistent with our hypothesis that areas with little exposure to medical cannabis prior to legalization would experience larger reductions in opioid-related outcomes as new recreational stores opened up, compared to counties which already had access to cannabis through the medical market.

The paper has several limitations. First, despite using sub-state data, our study may still suffer from aggregation bias given our focus on the county level. Second, our analysis ignores county-specific policies targeting the opioid epidemic that are time varying, such as differential distribution of naloxone or inappropriate prescribing campaigns. The exclusion of these local policies and campaigns should have biased results away from zero, if they were positively correlated with expansion of recreational cannabis markets. Third, while CHA represents nearly all Coloradans, there is variation in the hospitals and emergency departments reporting data to CHA. Moreover, the CHA outcomes used in this study reflect heavy or hazardous use of opioids sufficiently risky to generate a hospital or ED visit. Other margins of opioid use without a prescription or a physician’s supervision may not have the same relationship as those identified here.

Unfortunately, our findings do not clarify the muddy waters of the debate surrounding cannabis’s utility as a substitute for opioids and/or a treatment for opioid use disorder.^[26–28,43]^ Sales from the adult-use market exceeded that from the medical market before the end of the first year of recreational sales in 2014, and have only continued to grow at double digit rates since.^[44]^ However, we do not find similar blockbuster declines in opioid prescribing or opioid-related hospital visits. This suggests that while cannabis may have therapeutic benefits to patients suffering with particular ailments, it is not the panacea for solving the opioid crisis. Future work should examine changes in opioid utilization patterns using individual-level data to see if community exposure to cannabis changes opioid utilization among individuals, or among certain types of patients using opioids, such as the casual user versus the chronic pain patient.

## Data Availability

Outcome data used in this study are proprietary to the data set owners and are not available for use from the authors.
